# Use of Point-of-Care Ultrasound to Diagnose a Ruptured Splenic Hemangioma

**DOI:** 10.7759/cureus.63698

**Published:** 2024-07-02

**Authors:** Zachary A Glusman, Jeremy J Webb

**Affiliations:** 1 Emergency Department, LewisGale Medical Center, Salem, USA

**Keywords:** bedside ultrasound, point of care ultrasound, hemorrhagic shock, hemorrhagic splenic cyst, splenic cyst, rapid ultrasound in shock, rush exam

## Abstract

An 89-year-old female presented to the emergency department (ED) with hypotension and altered mental status. The patient had no external signs of trauma or hemorrhage and no abdominal tenderness on examination. The patient remained hypotensive after initial fluid resuscitation, and laboratory testing revealed a significant anemia. Point-of-care ultrasound (POCUS) was used to perform a rapid ultrasound in shock (RUSH) exam in an attempt to uncover the etiology of undifferentiated hypotension. The exam displayed free fluid in the right upper quadrant and the left upper quadrant exam demonstrated a large splenic lesion with mixed echogenicity. Subsequent computed tomography (CT) of the abdomen and pelvis with intravenous contrast suggested a ruptured hemorrhagic splenic cyst, and the patient underwent an emergent splenectomy for hemorrhage control. Operative pathologic examination revealed the cystic lesion to be a splenic hemangioma. This case report highlights the utility of the Rapid Ultrasound for Shock and Hypotension (RUSH) protocol when evaluating patients with undifferentiated nontraumatic shock, and a rare cause of spontaneous intra-abdominal hemorrhage.

## Introduction

The Rapid Ultrasound for Shock and Hypotension (RUSH) exam is an ultrasound protocol used for bedside investigation of undifferentiated hypotension [1-2]. The protocol includes views of the lungs, heart, aorta, inferior vena cava, and abdomen to search for reversible causes of shock such as pneumothorax, cardiac tamponade, aortic aneurysm rupture, intraperitoneal hemorrhage, and hypovolemia [1-2]. When performed and interpreted, this exam can provide clinicians with data that may lead to a rapid diagnosis [1-2]. Ultrasonography, when performed by experienced users, can have a high sensitivity and specificity for detecting evidence of hypovolemic shock. One meta-analysis reported a sensitivity of 0.87 and specificity of 0.98 for all types of shock, with sensitivity ranging from 0.91 to 1.00 and specificity from 0.87 to 0.98 specifically for hypovolemic shock [1]. Subgroup analysis in another study comparing emergency medicine physicians to radiologists showed a kappa index of 0.70 and 0.73, respectively [2], indicating good interrater reliability. Therefore, the RUSH exam can be an important bedside adjunct in the emergency department (ED) to promptly identify sonographic signs of shock in non-trauma patients.

## Case presentation

An 89-year-old female presented to the ED with hypotension and altered mental status. The family reported her blood pressure (BP) was less than 90/60 for the entire day, and she had altered mental status. The family denied any recent trauma but did report recent severe, watery, non-bloody diarrhea. This was suspected by the family to be the result of recent laxative use taken for constipation. Past medical history included orthostatic hypotension, anemia, and a previous stroke with no residual focal deficits. Baseline mental status was alert and oriented to person and place. The patient was walker dependent, but the family reported increasing difficulty with ambulation and activities of daily living (ADLs) over the past few months. Her daily medications consisted of aspirin 81 mg and midodrine only, and she had not missed any doses.

The patient was mildly somnolent on arrival, confused, and followed limited commands with a bilateral hand squeeze. Initial vitals showed a BP of 90/55 mmHg, a heart rate of 75 beats per minute, and pulse oximetry of 99% on room air. Cardiac and pulmonary examinations were unremarkable. The patient’s abdomen was soft and non-tender. Subsequent systolic BP after a 1-L bolus of normal saline remained at 90. Non-contrast head computed tomography (CT) was unremarkable. Complete blood count (CBC) showed anemia, with hemoglobin (Hgb) of 6.1 g/dL and the mean corpuscular volume (MCV) of 100.5 fL. The most recent Hgb value one year prior was 8.8 g/dL. Two units of packed red blood cells were transfused. The laboratory fecal occult blood test (FOBT) was negative. 

**Figure 1:**
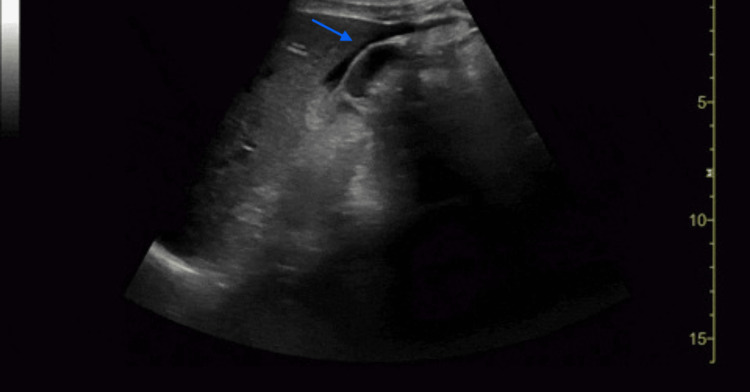
RUQ abdominal ultrasound with free fluid Anechoic fluid is identified by the blue arrow near the edge of the liver. RUQ, right upper quadrant

**Figure 2:**
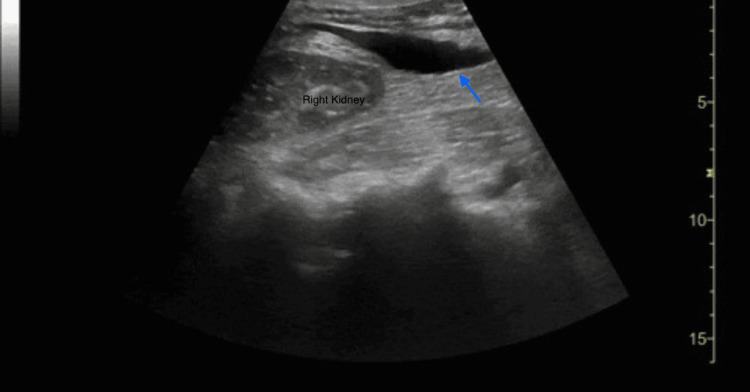
RUQ abdominal ultrasound near the caudal liver tip with free fluid. Anechoic fluid identified by the blue arrow just past the caudal tip of the liver and adjacent to the right kidney. RUQ, right upper quadrant

**Figure 3:**
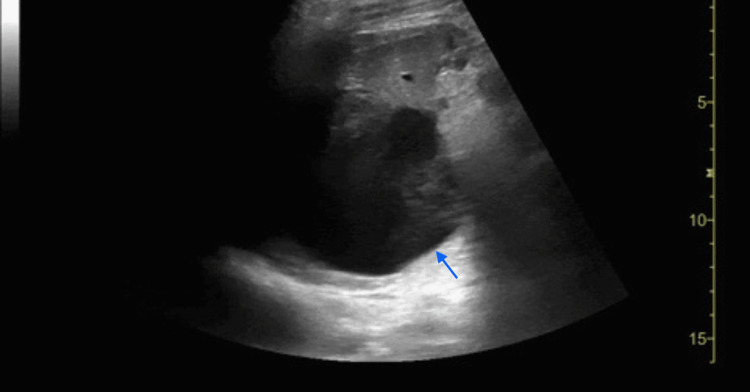
LUQ abdominal ultrasound showing a large cystic structure within the spleen. The cystic structure is identified in the spleen by a blue arrow. LUQ, left upper quadrant

**Figure 4:**
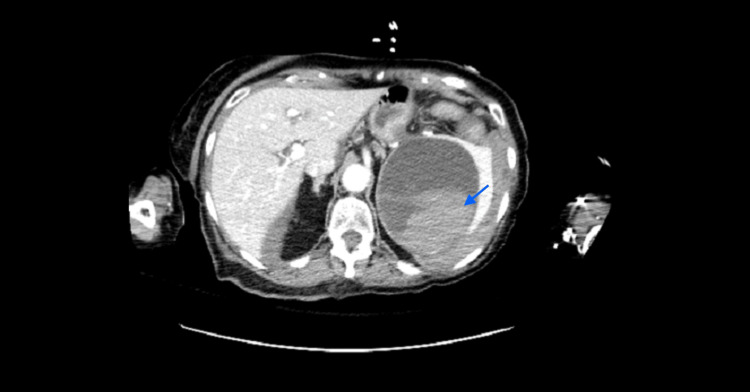
Axial abdominal CT imaging showing the spleen with a patchy splenic hyperdensity and dense perisplenic fluid. Splenic lesion identified by a blue arrow. CT, computed tomography

**Figure 5:**
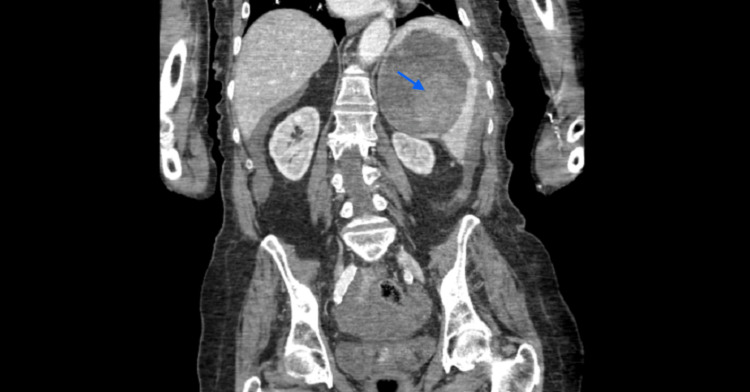
Coronal abdominal CT imaging showing a ruptured splenic hemangioma. Splenic lesion Identified by a blue arrow.

Due to continued undifferentiated hypotension, point-of-care ultrasound (POCUS) was used to perform a RUSH exam. The exam revealed free fluid in the right upper quadrant, and the left upper quadrant exam demonstrated a large cystic lesion within the spleen with mixed echogenicity (Figures 1-3). Subsequent abdominopelvic CT with intravenous contrast showed free fluid in the abdomen and pelvis with a large complex cystic lesion in the spleen (Figures 4-5). These findings were most consistent with a ruptured hemorrhagic splenic cyst according to the radiology report. General surgery was consulted, and the patient was taken expeditiously to the operating room for exploratory laparotomy and splenectomy. Subsequent pathology reports diagnosed the splenic lesion as a ruptured splenic hemangioma. After an uncomplicated short course in the hospital, the patient was discharged to home at her baseline mental status.

## Discussion

This case outlines the limitations of history and physical examination in an altered patient with undifferentiated shock. On initial evaluation, volume loss through diarrhea and laxative use was presumed to be the origin of hypotension. However, the patient remained hypotensive after fluid resuscitation. Lab testing revealed an anemia that could have represented hemorrhagic shock, but there was no identifiable source. There was no reported hematemesis or bloody stool, and a negative FOBT made gastrointestinal bleeding less likely. The abdominal exam also had no signs of peritonitis. A RUSH examination was performed to evaluate for an etiology of hypotension. This ultimately led to the diagnosis of a ruptured hemorrhagic splenic hemangioma. 

Splenic hemangiomas are the most common benign neoplasm of the spleen [3-5]. However, they are still quite rare with an estimated incidence between 0.03% and 14% based off of autopsy series [3-5]. Rupture of these lesions is also rare as one case series reported rupture occurring in 1 of 31 patients (3.2% risk) [6]. Ultrasound appearance can vary widely from predominately being solid, mixed, or a pure cystic lesion [7]. Hemangiomas typically have a periphery and a hypoechoic center [7]. Atypical characteristics such as heterogenous echogenicity with hypoechoic areas due to necrosis, hemorrhage, and thrombosis are common in larger lesions [7]. These characteristics illustrate the discrepancies between our imaging and pathology results as a splenic cyst can appear similar to a splenic hemangioma. While the literature is conflicting on treatment for small or asymptomatic splenic hemangiomas, splenectomy and/or splenic artery embolization is typically advised when ruptured or symptomatic [8-9]. 

In the ED, it can be challenging to diagnose the etiology of undifferentiated shock based solely on clinical evaluation and physical examination. One study found that standard physical examination identified a correct cause of shock in only 45% of cases [10]. However, when the standard examination was combined with POCUS, 89% of patients were diagnosed with the correct corresponding etiology of shock [10]. Another study showed similar results, with the RUSH exam demonstrating 86% diagnostic accuracy [11]. 

The performance of a RUSH examination has been shown to improve time-to-diagnosis, particularly in resource-limited environments. One study showed the RUSH protocol was able to diagnose the cause of shock in an average of 12 minutes without interrupting the ongoing resuscitation [11]. In contrast, obtaining a CT scan will interrupt resuscitation and may be dangerous in an unstable patient. In this patient's case, we initially did not indicate to obtain a CT scan. Before the performance of the RUSH exam, the plan was to continue with fluid resuscitation as hypovolemic shock secondary to diarrhea was expected. The RUSH exam showed signs of hemorrhagic shock with a splenic source. Due to patient stability, this was confirmed with CT imaging for operative planning. Without the performance of the RUSH exam, we surmise the diagnosis would have been delayed and could have resulted in significant morbidity or mortality. We recommend continued use of the RUSH exam by Emergency Medicine (EM) physicians to rapidly diagnose patients with undifferentiated non-traumatic hypotension. 

## Conclusions

POCUS is a rapidly growing resource for EM physicians. EM physicians have proven to adequately perform and interpret POCUS imaging. In this case, POCUS expedited emergent surgery that ultimately led to the patient’s safe discharge. The RUSH exam has been shown to adequately diagnose the type of shock in an expedited timeframe, and result in more efficient management. EM physicians should consider adding the RUSH exam as an adjunct exam in all of their undifferentiated shock patients. 
